# Bilateral Hypertrophy of the m. Tensor Fascia Latae

**DOI:** 10.5334/jbsr.2724

**Published:** 2022-05-09

**Authors:** Cedric De Clercq, Lennart Jans, Koenraad Verstraete

**Affiliations:** 1 University of Ghent, BE; 2 Ghent University Hospital, BE; 3 Academic Chief Department of Radiology Ghent University Hospital and Ghent University, BE

**Keywords:** pseudotumor, TFL, hypertrophy, tensor fascia latae, MRI

## Abstract

**Teaching point:** Hypertrophy of the m. tensor fascia lata mimics a soft tissue tumor, but understanding of its presentation on MRI prevents unnecessary biopsy.

## Case

A 62-year-old woman presented with a swelling on the proximal aspect of the thigh. The patient stated the swelling started several weeks ago and increased ever since. The patient had an unremarkable medical history and did not present with fever, weight loss, night sweats, or disturbed sleep. She stated that she had no pain when walking, but the pain was present when she laid on the left thigh.

Physical examination showed a swelling of the proximal thigh, just below the anterior superior iliac spine, which was not painful on palpation.

**Figure 1:**
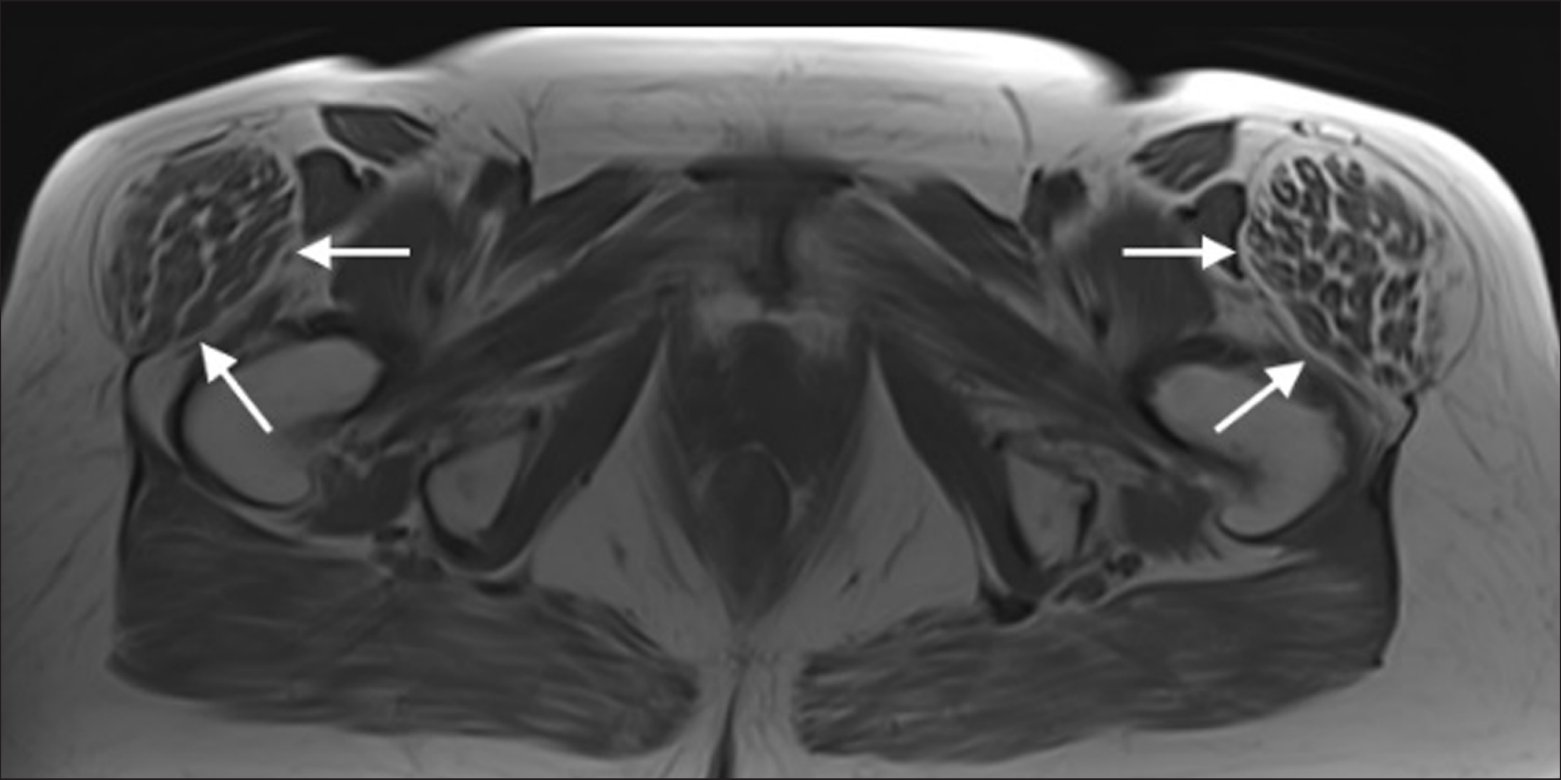


**Figure 2:**
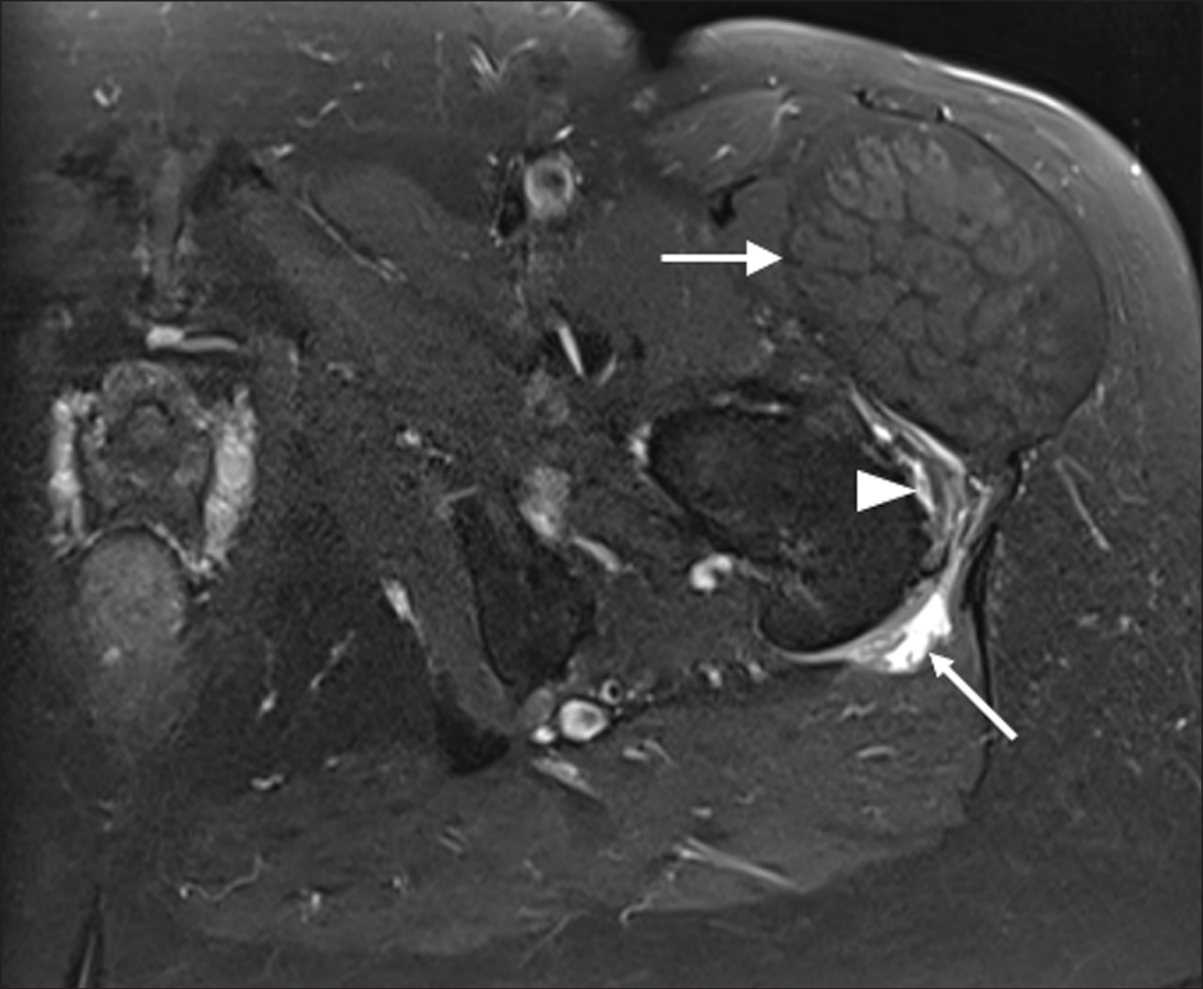


**Figure 3:**
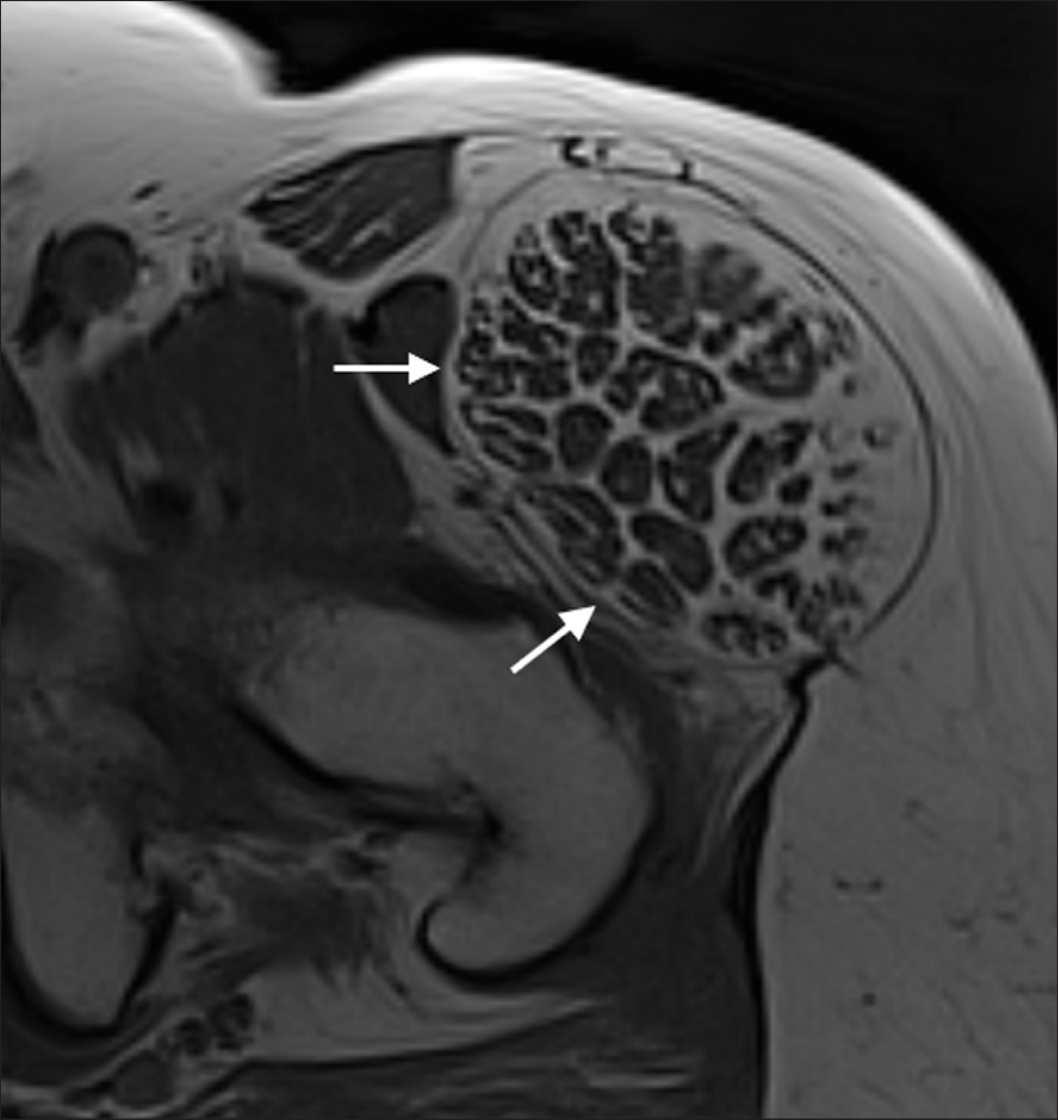


The patient was referred to the radiology department for magnetic resonance imaging (MRI). The T1-weighted images showed bilateral hypertrophic tensor fasciae latae (TFL) muscles (***Figures 1*** and ***3***, arrows). This was more prominent on the left side. Besides hypertrophy, the images showed fatty degeneration of the muscle. The T2-weighted images with fat suppression showed neither oedema of the muscle belly, nor soft tissue inflammation. However, on the left side, there was an enlarged trochanteric bursa (***Figure 2***, small arrow) consistent with bursitis, and both a tear of the m. gluteus minimus tendon (***Figure 2***, arrowhead) and tendinosis of the m. gluteus medius.

## Comment

A palpable soft tissue mass is a common problem in clinical practice. It has a broad differential diagnosis which includes neoplasms, hematomas, and hypertrophied muscle. Due to the possibility of malignancy, further investigation is mandatory. MRI is the first-choice imaging technique, due to its superior soft tissue contrast and multiplanar image capability [1].

Common etiologies of muscle hypertrophy include: exercise, denervation, radiation, and myopathies. Muscle hypertrophy of the TFL is rather uncommon, with only case series published including three patients and reporting biopsy-proven muscle hypertrophy in three patients with concomitant necrotizing myopathy and non-inflammatory myopathy in one patient respectively [1]. In our patient, no biopsy was performed.
